# The effect of Δ9-tetrahydrocannabinol, cannabidiol, menthol and propofol on 5-hydroxytryptamine type 3 eeceptors--a computational approach

**DOI:** 10.1186/1471-2202-16-S1-P290

**Published:** 2015-12-18

**Authors:** Andreas C Schilbach, Tatiana Prytkova, Susan Keun-Hang Yang

**Affiliations:** 1 Schmid College of Science and Technology, Chapman University, Orange CA, 92866, USA

## 

This study investigates the function of 5-HT type 3 (5-HT3) receptors using a computational approach. Antagonists of the 5-HT3 receptor are currently one of the most effective therapeutic agents in treatment of chemotherapy-induced nausea, vomiting, and irritable bowel syndrome. Several experimental studies have shown the effect of pharmacological agents such as 9-tetrahydrocannabinol (THC), the psychoactive component of Cannabis, Cannabidiol (CBD), a non-psychoactive ingredient of Cannabis plant, Menthol, Propofol, and etc. on the functional human 5-HT3 receptors expressed in Xenopus oocyte as well as rat nodose ganglion neurons [1,2]. 5-HT evoked currents recorded by a two-electrode clamp technique were inhibited by ligands in a concentration dependent manner. Simulations of allosteric inhibition were modeled using Vina docking techniques with the 5-HT3 structure (see Tables 1 and 2 for results). The 5-HT3 structure was found using homology sequence similarity techniques with the neuronal nicotinic acetylcholine receptor (nACH) and inhibitory neurotransmitter receptor for GABA(A). Results of studies with other members of the superfamily of ligand gated ion channels signified key residues involved in ligand binding sites within the transmembrane region of 5-HT3 [3]. Flexible and rigid docking simulations around key residues resulted in a number of low-energy (high affinity) configurations of ligand binding (Figure 2). The predicted residues TYR and THR may constitute a naturally occurring binding site for 5-HT3.

**Table 1:** Docking of Ligands on lower (THR361) binding sites of 5-HT3A

Compound - (THR361 binding site)	Binding energy (kcal/mol)
Cyclohexane	-4.5

Eucalyptol	-4.5

Menthol	-5.6

Propofol	-6.0

**Table 2:** Docking of Ligands on upper (TYR346) binding sites of 5-HT3A

Compound - (TYR346 binding site)	Rigid Docking - Binding energy (kcal/mol)	Flexible Docking - Binding energy (kcal/mol)
Δ9-tetrahydrocannabinol (THC)	-7.0	-9.2

Cannabidiol (CBD)	-7.0	-8.8

**Figure 1:**
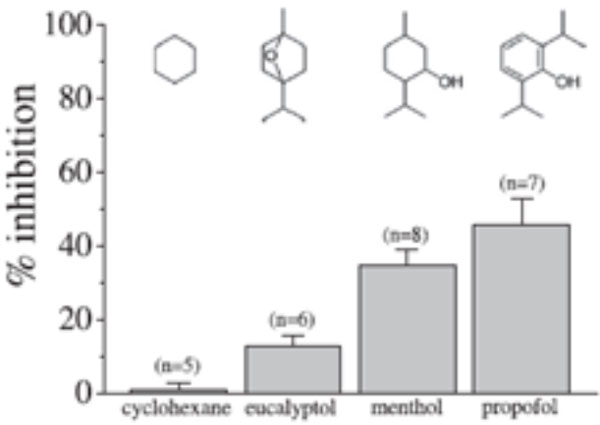
**Experimental comparison **[3]**of the effects of compounds structurally related to menthol on the 5-HT3 receptors**.

**Figure 2:**
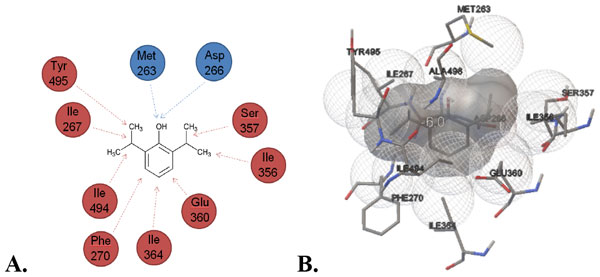
**Interaction diagrams of Propofol with lower binding site (THR361) of 5-HT3A**. **A**. 2-D Ligand interaction diagram. Residues are annotated with their 3-letter amino acid code, and their position classification. Hydrophilic interactions: include the hydroxyl group with residue Asp266 and Met263 (Blue). Hydrophobic interactions: Ile (267, 356, 364, 494), Ser357, Glu360, and Tyr 495 (Red) **B**. 3-D depiction of interaction.

## Conclusion

Experimental inhibition of 5-HT3 shows similar trend in computational binding energies to the lower binding site (THR 361).Docking calculations provide explanation of molecular basis of difference in inhibition by menthol like compounds. Similar binding energies for THC and CBD corresponds to their similar inhibition of membrane currents measured in experiment.
